# Supplementary material for: HIVprotI: an integrated web based platform for prediction and design of HIV proteins inhibitors
Source: J Cheminform. 2018 Mar 9;10:12. doi: 10.1186/s13321-018-0266-y (PMC5845081; doi:10.1186/s13321-018-0266-y)
Supplement: Supplementary file 2 — Additional file 2. Source code of HIVProtI web server. [file 13321_2018_266_MOESM2_ESM.zip › HIVprotI_Source-code/hivprotI/weka-3-6-6/doc/weka/experiment/package-tree.html]

weka.experiment Class Hierarchy


---


|  |  |  |
| --- | --- | --- |
| **Overview** \| **Package** \| Class \| **Tree** \| **Deprecated** \| **Index** \| **Help** \| **Weka's home** | |  |
| **PREV**   **NEXT** | **FRAMES**    **NO FRAMES**     **All Classes** |  |


---


## Hierarchy For Package weka.experiment

**Package Hierarchies:**: All Packages

---

## Class Hierarchy

- java.lang.Object
  - weka.experiment.**AveragingResultProducer** (implements weka.core.AdditionalMeasureProducer, weka.core.OptionHandler, weka.experiment.ResultListener, weka.experiment.ResultProducer, weka.core.RevisionHandler)- weka.experiment.**ClassifierSplitEvaluator** (implements weka.core.AdditionalMeasureProducer, weka.core.OptionHandler, weka.core.RevisionHandler, weka.experiment.SplitEvaluator)
      - weka.experiment.**CostSensitiveClassifierSplitEvaluator**
      - weka.experiment.**CrossValidationResultProducer** (implements weka.core.AdditionalMeasureProducer, weka.core.OptionHandler, weka.experiment.ResultProducer, weka.core.RevisionHandler)- weka.experiment.**CSVResultListener** (implements weka.core.OptionHandler, weka.experiment.ResultListener, weka.core.RevisionHandler)
          - weka.experiment.**InstancesResultListener**
          - weka.experiment.**DatabaseUtils** (implements weka.core.RevisionHandler, java.io.Serializable)
            - weka.experiment.**DatabaseResultListener** (implements weka.experiment.ResultListener)
              - weka.experiment.**DatabaseResultProducer** (implements weka.core.AdditionalMeasureProducer, weka.core.OptionHandler, weka.experiment.ResultProducer)
              - weka.experiment.**InstanceQuery** (implements weka.core.OptionHandler)
            - weka.experiment.**DensityBasedClustererSplitEvaluator** (implements weka.core.AdditionalMeasureProducer, weka.core.OptionHandler, weka.core.RevisionHandler, weka.experiment.SplitEvaluator)- weka.experiment.**Experiment** (implements weka.core.OptionHandler, weka.core.RevisionHandler, java.io.Serializable)
                - weka.experiment.**RemoteExperiment**
                - weka.experiment.**LearningRateResultProducer** (implements weka.core.AdditionalMeasureProducer, weka.core.OptionHandler, weka.experiment.ResultListener, weka.experiment.ResultProducer, weka.core.RevisionHandler)- weka.experiment.**OutputZipper** (implements weka.core.RevisionHandler)- weka.experiment.**PairedStats** (implements weka.core.RevisionHandler)
                      - weka.experiment.**PairedStatsCorrected**
                      - weka.experiment.**PairedTTester** (implements weka.core.OptionHandler, weka.core.RevisionHandler, weka.experiment.Tester)
                        - weka.experiment.**PairedCorrectedTTester** (implements weka.core.TechnicalInformationHandler)
                        - weka.experiment.**PropertyNode** (implements weka.core.RevisionHandler, java.io.Serializable)- weka.experiment.**RandomSplitResultProducer** (implements weka.core.AdditionalMeasureProducer, weka.core.OptionHandler, weka.experiment.ResultProducer, weka.core.RevisionHandler)- weka.experiment.**RegressionSplitEvaluator** (implements weka.core.AdditionalMeasureProducer, weka.core.OptionHandler, weka.core.RevisionHandler, weka.experiment.SplitEvaluator)- weka.experiment.**RemoteExperimentEvent** (implements java.io.Serializable)- weka.experiment.**RemoteExperimentSubTask** (implements weka.core.RevisionHandler, weka.experiment.Task)- java.rmi.server.RemoteObject (implements java.rmi.Remote, java.io.Serializable)
                                    - java.rmi.server.RemoteServer
                                      - java.rmi.server.UnicastRemoteObject
                                        - weka.experiment.**RemoteEngine** (implements weka.experiment.Compute, weka.core.RevisionHandler)
                                    - weka.experiment.**ResultMatrix** (implements weka.core.RevisionHandler, java.io.Serializable)
                                      - weka.experiment.**ResultMatrixCSV**- weka.experiment.**ResultMatrixGnuPlot**- weka.experiment.**ResultMatrixHTML**- weka.experiment.**ResultMatrixLatex**- weka.experiment.**ResultMatrixPlainText**- weka.experiment.**ResultMatrixSignificance**
                                      - weka.experiment.**Stats** (implements weka.core.RevisionHandler, java.io.Serializable)- weka.experiment.**TaskStatusInfo** (implements weka.core.RevisionHandler, java.io.Serializable)

## Interface Hierarchy

- java.rmi.Remote
  - weka.experiment.**Compute**
  - weka.experiment.**RemoteExperimentListener**- java.io.Serializable
      - weka.experiment.**ResultListener**- weka.experiment.**ResultProducer**- weka.experiment.**SplitEvaluator**- weka.experiment.**Task**- weka.experiment.**Tester**

---


|  |  |  |
| --- | --- | --- |
| **Overview** \| **Package** \| Class \| **Tree** \| **Deprecated** \| **Index** \| **Help** \| **Weka's home** | |  |
| **PREV**   **NEXT** | **FRAMES**    **NO FRAMES**     **All Classes** |  |


---
